# Comparison of structural variants detected by PacBio-CLR and ONT sequencing in pear

**DOI:** 10.1186/s12864-022-09074-7

**Published:** 2022-12-14

**Authors:** Yueyuan Liu, Mingyue Zhang, Runze Wang, Benping Li, Yafei Jiang, Manyi Sun, Yaojun Chang, Jun Wu

**Affiliations:** 1 State Key Laboratory of Crop Genetics and Germplasm Enhancement, College of Horticulture, Nanjing Agricultural University, Nanjing, 210095 Jiangsu China; 2 College of Horticultural Science and engineering, Shandong Agricultural University, Taian, 271018 Shandong China; 3 Novogene Bioinformatics Institute, Beijing, China

**Keywords:** Asian and European pear, PB-CLR sequencing, ONT sequencing, SV detection

## Abstract

**Background:**

Structural variations (SVs) have recently become a topic of great interest in the area of genetic diversity and trait regulation. As genomic sequencing technologies have rapidly advanced, longer reads have been used to identify SVs at high resolution and with increased accuracy. It is important to choose a suitable sequencing platform and appropriate sequencing depth for SV detection in the pear genome.

**Results:**

In this study, two types of long reads from sequencing platforms, continuous long reads from Pacific Biosciences (PB-CLR) and long reads from Oxford Nanopore Technologies (ONT), were used to comprehensively analyze and compare SVs in the pear genome. The mapping rate of long reads was higher when the program Minimap2 rather than the other three mapping tools (NGMLR, LRA and Winnowmap2) was used. Three SV detection programs (Sniffles_v2, CuteSV, and Nanovar) were compared, and Nanovar had the highest sensitivity in detecting SVs at low sequencing depth (10–15×). A sequencing depth of 15× was suitable for SV detection in the pear genome using Nanovar. SVs detected by Sniffles_v2 and CuteSV with ONT reads had the high overlap with presence/absence variations (PAVs) in the pear cultivars ‘Bartlett’ and ‘Dangshansuli’, both of them with 38% of insertions and 55% of deletions overlapping with PAVs at sequencing depth of 30×. For the ONT sequencing data, over 37,526 SVs spanning ~ 28 Mb were identified by all three software packages for the ‘Bartlett’ and ‘Dangshansuli’ genomes. Those SVs were annotated and combined with transcriptome profiles derived from ‘Bartlett’ and ‘Dangshansuli’ fruit flesh at 60 days after cross-pollination. Several genes related to levels of sugars, acid, stone cells, and aromatic compounds were identified among the SVs. Transcription factors were then predicted among those genes, and results included *bHLH, ERF,* and *MYB* genes.

**Conclusion:**

SV detection is of great significance in exploring phenotypic differences between pear varieties. Our study provides a framework for assessment of different SV software packages and sequencing platforms that can be applied in other plant genome studies. Based on these analyses, ONT sequencing data was determined to be more suitable than PB-CLR for SV detection in the pear genome. This analysis model will facilitate screening of genes related to agronomic traits in other crops.

**Supplementary Information:**

The online version contains supplementary material available at 10.1186/s12864-022-09074-7.

## Introduction

Structural variation (SV) is generally defined as a large-scale structural difference in a region of genomic DNA that is inherited and polymorphic [1]. SV types include insertion (INS), deletion (DEL), inversion (INV), duplication (DUP), and translocation (TRA)/breakend (BND) [2]. Structural variation studies in plants are increasingly common and have been applied to understand genomic changes during evolution, domestication, and breeding [3]. Recently, several pan-genome studies have been conducted in different plant species, and presence/absence variation (PAV) diversity has been investigated [4].

Previous studies have shown that SVs can directly affect crop phenotypes such as fruit yield and quality [3]. It is now highly feasible to identify the extent and impact of SVs in crop genomes as genome sequencing technologies continue to evolve, especially as it becomes easier to produce accurate long sequence reads at a genome-wide scale [3]. In grapes, strong purifying selection acts on SVs, especially inversions and translocations. SVs accumulate as recessive heterozygotes in clonal lineages [5]. Sicilian blood oranges originated from the insertion of a Copia-like retrotransposon adjacent to the gene encoding Ruby, a MYB transcriptional activator of anthocyanin production [6]. In the *Pyrus betuleafolia* (*Pbe-SD*) genome, an insertion SV was identified in the promoter region of *barely any meristem 1* (*BAM1*, Chr12.g42983), which may influence the morphogenesis and cell differentiation of plant meristems [7]. In the ‘Zaosu red’ pear (*Pyrus*) cultivar, a 14-bp nucleotide deletion in the coding region of the *PpBBX24* gene was identified and may be associated with the red skin trait [8]. It remains unclear whether agronomic trait differences between Asian and European pear varieties are related to SVs.

Pear is an economically important fruit tree grown worldwide, with thousands of cultivars of five domesticated (and dozens of wild) species [9]*.* Asian and European pears show distinct phenotypic traits [9]. European pears are typically pear-shaped fruits with soft, smooth flesh, few stone cells, and strong aroma and flavor, whereas Asian pears are round fruits with crisp flesh, high sugar content, low acid content, minimal aroma, and mild flavor [9]. Reference genomes were assembled for the cultivars ‘Dangshansuli’ and ‘Bartlett’ in 2013 and 2014, respectively [10, 11]. The ‘Bartlett’ genome (BartlettDHv2.0) was improved using a combination of PacBio RSII long-read sequencing, Bionano optical mapping, chromatin interaction capture (Hi-C), and genetic mapping in 2019 [12]. The SVs responsible for the observed phenotypic differences in fruit traits between Asian and European pears have not been well explored.

Pacific Biosciences® SMRT Sequencing (PacBio) [13] and Oxford Nanopore sequencing (ONT) [14] can both produce long reads (> 10 kb), giving them an advantage in comprehensive detection of SVs because they can span repetitive or other faulty regions [15]. For PB-CLR (Pacbio-CLR), the N50 read length from platform Sequel II was 30–60 kb, the maximum of read length was over 200 kb. The average throughput per flow cell was 50–100 Gb and the estimated cost per Gb was $13–26. The read accuracy was 87–92%. For ONT, the N50 read length from platform PromethION was 10–60 kb, the maximum of read length was over 1000 kb. The average throughput per flow cell was 50–100 Gb and the estimated cost per Gb was $21–42. The read accuracy was 87–98% [16]. To account for the increased error rate of these long-read sequencing technologies, new alignment tools have been developed, such as Minimap2 [17], NGMLR [18] and LRA [19]. The aligner Winnowmap2 was developed for more sensitive SV detection in repeat sequences [20].

There are several software packages designed to detect SVs from both Pacbio and ONT reads. Sniffles is a read-alignment-based SV detector. It can use both within-alignment and split-read information to detect SVs; small insertions/deletions (InDels) can be found within a single alignment, whereas large or complex events lead to split-read alignments. Sniffles_v1 excels at filtering false SV signals from noisy reads [18]. Sniffles_v1 (v1.0.11) [18] was reported to detect SVs with higher accuracy than another tool, SVIM [21], using PacBio sequencing data for the ‘Yali’ pear genome [22]. Furthermore, the latest version of Sniffles_v2 (v2.0.6) is capable of detecting more SVs across different coverages (10.1101/2022.04.04.487055). CuteSV is also a read-alignment-based SV detection tool. CuteSV has high SV detection sensitivity, especially for lower-coverage datasets, and it can achieve a nearly linear multiple-thread speed during data processing [23]. Nanovar was developed for SV detection in low-depth ONT data from human patients. It uses a neural-network-based algorithm for high-confidence detection and zygosity estimation of all SV classes [24].

It is important to identify an appropriate sequencing depth for long-read platforms to allow optimal allocation of limited resources. Human genomes have been used to compare the number of SVs detected and the recall rate at different sequencing depths using both PacBio and ONT data; a depth of 10× was sufficient to infer SV breakpoints, but increased sequencing depth was associated with a higher recall rate [18]. The impact of different sequencing depths on SV detection in long-read sequencing data remains unclear. A next-generation sequencing depth of 50× is appropriate for detecting SVs in the pear genome [22]. However, the most suitable sequencing depth for detecting SVs from long reads has not yet been determined in pear.

We here evaluated the effectiveness of three SV detection software packages (Sniffles_v2, CuteSV, and Nanovar) on *Pyrus* data derived from two long-read sequencing platforms (PB-CLR and ONT). The Asian pear cultivar selected was ‘Dangshansuli’ (*P. bretschneideri*), which is one of the primary pear cultivars grown in China. The reference genome used was the updated release of the European pear ‘Bartlett’ (*P. communis*), BartlettDHv2.0 [12]. We conducted a systematic analysis starting with four different mapping tools, Minimap2 [17], NGMLR [18], LRA [19] and Winnowmap2 [20]. The effects of different sequencing depths and long-read platforms on SV detection were investigated, and the most appropriate sequencing depth for detecting SVs in *Pyrus* was determined by comparing the number and precision of SVs detected. Furthermore, we investigated the SVs identified by all three tested SV detection programs and compared those identified from PB-CLR and from ONT sequencing data. The identified SVs were then annotated, and differentially expressed genes (DEGs) within the SVs were determined by comparing transcripts between ‘Bartlett’ and ‘Dangshansuli’ fruit flesh. DEGs were screened using GO terms and KEGG pathway annotations to identify genes that potentially affect differing agronomic traits between Asian and European pear. Our findings lay the foundation for subsequent studies of SVs in other crop species; the pipeline constructed for this study can be used to explore genes within SVs that may cause differences in agronomic traits between varieties of crop species.

## Results

### Long-read sequencing and mapping of ‘Dangshansuli’ pear

Long-read sequencing data was generated for ‘Dangshansuli’ using the PB-CLR and ONT platforms. The resulting sequencing depth for both platforms was 30×. After filtering out low quality reads, a total of 1,002,476 PB-CLR subreads were obtained. The average subreads length was over 20 kb and the N50 was > 27 kb. For the ONT data, a total of 907,633 reads with a mean read length over 18 kb were obtained, and the N50 length was > 18 kb. Seqtk was used to randomly extract sequences to different depths (10, 15, 20, 25, and 30× coverage). The size of ‘Bartlett’ pear genome is 528 Mb [12]. All of the resulting sequencing reads from both PB-CLR and ONT were mapped to the ‘Bartlett’ reference genome using Minimap2 [17], NGMLR [18], LRA [19] and Winnowmap2 [20].

**Fig. 1:**
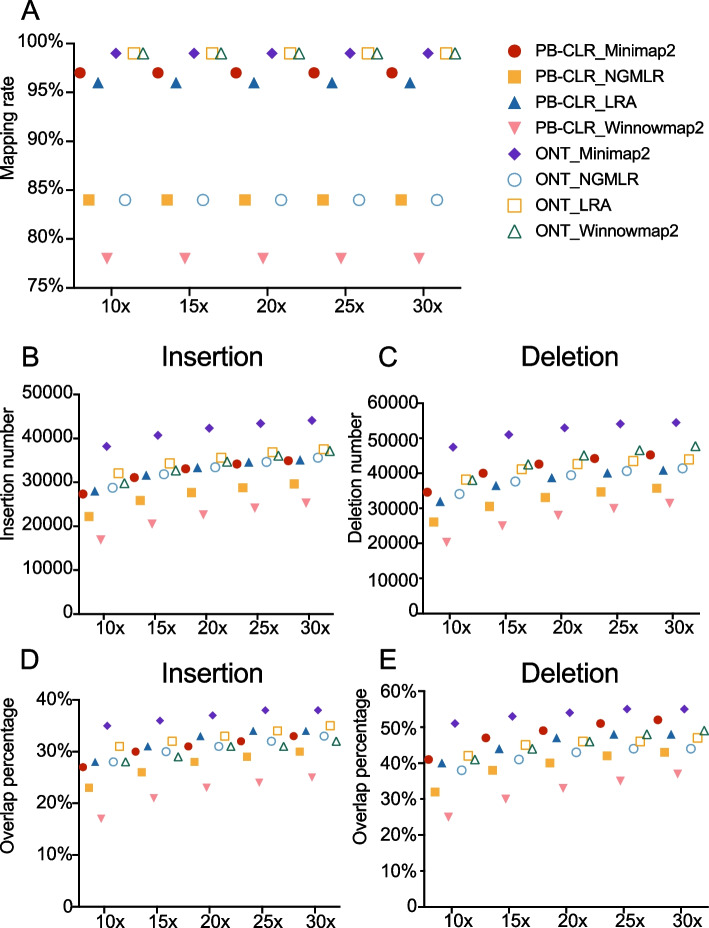
Comparison of four mapping tools (Minimap2, NGMLR, LRA and Winnowmap2) performance on PacBio and ONT sequencing data. **A** The mapping rate of four mapping tools on PB-CLR and ONT sequencing data. **B**, **C** The number of insertions (**B**) and deletions (**C**) detected by Sniffles_v2 after using four mapping tools on PB-CLR and ONT sequencing data. **D** The percentage of SVs that overlapped with presence/absence variations (PAVs) between the pear cultivars ‘Bartlett’ and ‘Dangshansuli’ using four mapping tools on PB-CLR and ONT sequencing data

Among the four aligners, Minimap2 has the highest mapping rate on both PB-CLR and ONT data across different sequencing coverages (10×, 15×, 20×, 25× and 30×) (Fig. 1). For Minimap2, the primary mapping rate ranged from 97.41–99.30%; the range was 84.13–84.44% for NGMLR. For Minimap2, the mapping rate was higher for ONT (99.28–99.30%) than for PB-CLR sequencing data (97.41–97.46%) (Fig. 1A). The mapping rate level of LRA was second only to Minimap2, which was for PB-CLR data (96.17–96.25%) and for ONT data (99.09–99.12%). The mapping rate for NGMLR was higher for PB-CLR (84.40–84.43%) than ONT sequencing data (84.13–84.17%). The mapping rate of Winnowmap2 with PB-CLR and ONT data showed significant difference, for PB-CLR is range from 77.63–77.83% while for ONT is range from 98.88–98.89%.

Three SV detectors (Sniffles_v2, CuteSV, and Nanovar) were used for SV calling after mapping with the four aligners. More insertions and deletions were identified when Minimap2 rather than the others was used for mapping (Fig. 1B&C, Additional file 1). When insertions and deletions were compared with PAVs identified between the ‘Bartlett’ and ‘Dangshansuli’ genomes, there was more overlap between SVs and PAVs when Minimap2 was used for mapping instead of the others (Fig. 1D). This established Minimap2 as a more suitable mapping tool, and Minimap2 was therefore used exclusively in further analyses.

### SV detection using three software packages on PB-CLR and ONT sequencing data

We benchmarked the SV calling performance of three state-of-the-art SV detection programs on the datasets at 10, 15, 20, 25, and 30× sequencing depth. The programs tested were Sniffles_v2, CuteSV, and Nanovar. This analysis was designed to evaluate the SV detection capabilities of each program at different sequencing depths on both PB-CLR and ONT sequencing data.

**Fig. 2:**
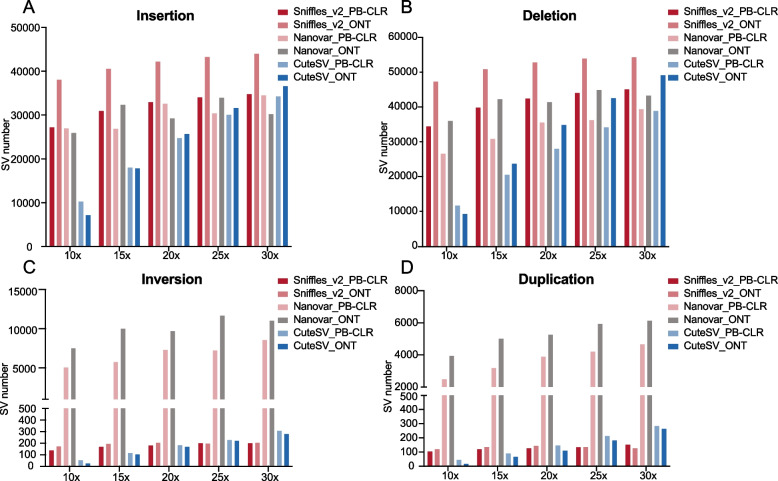
The number of SVs detected by three software packages (CuteSV, Nanovar, and Sniffles_v2) using PB-CLR and ONT data at a range of sequencing depths

To quantify the performance of the three SV callers, we counted four main types of SVs: insertions, deletions, inversions, and duplications. The distributions of SVs detected by the SV callers from PB-CLR and ONT sequencing data at different sequencing depths are shown Fig. 2. Out of all combinations of SV callers and sequencing platforms, the highest number of SVs were detected using Nanovar on ONT sequencing data. Furthermore, at low sequencing depths, Nanovar detected more SVs than the other software packages did and Sniffles_v2 can detect more insertion and deletions than the others (Fig. 2).

**Fig. 3:**
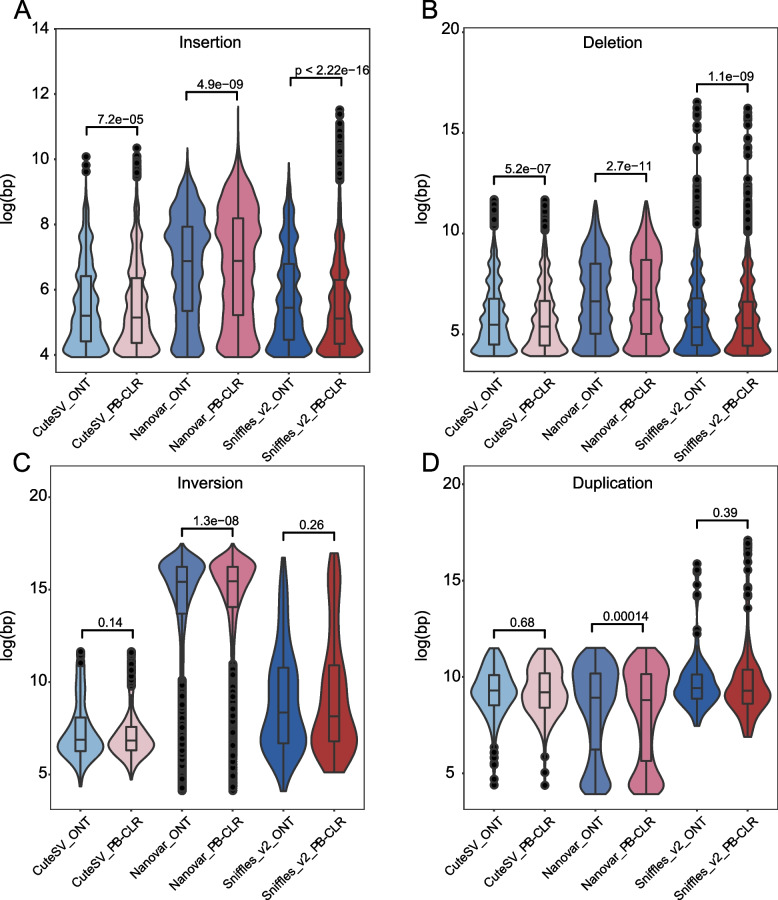
Comparison of the average lengths of insertions, deletions, inversions, and duplications detected by three software packages (CuteSV, Nanovar, and Sniffles_v2) from PB-CLR and ONT sequencing data

In the pear genome, the number of insertions and deletions detected by Sniffles_v2 in ONT data were highest (Fig. 2A&B). Sniffles_v2 detected more SVs with ONT data than with PB-CLR data. Sniffles_v2 could detect longer duplications (log(bp) > 15) than the other two SV calling programs (Fig. 3C).

CuteSV also called more insertions from the PB-CLR than the ONT data at low sequencing depths (10 and 15×). As the sequencing depth increased, CuteSV identified more insertions and deletions from ONT than PB-CLR data. There were slightly more inversions and duplications called by CuteSV from PB-CLR data than from ONT data. However, when the sequencing depth reached 30×, CuteSV called a bit more insertions and deletions than Nanovar (Fig. 2B). Among the three software packages, CuteSV identified deletions and inversions in PB-CLR sequencing data with the shortest average length (Fig. 3C&D).

Compared to Sniffles_v2 and CuteSV, Nanovar has superior sensitivity in detecting SVs at low sequencing depth. The number of SVs detected by Sniffles_v2 and Nanovar did not significantly increase with increased sequencing depth. Nanovar called far more inversions and duplications than Sniffles_v2 and CuteSV did (Fig. 2C&D). The average lengths of insertions, deletions, and inversions identified by Nanovar were the longest of the three software packages (Fig. 3A–C). Nanovar was found to detect more insertions and inversions with large segments than the other two programs did.

### SVs overlapping with PAVs between the ‘Dangshansuli’ and ‘Bartlett’ genomes

We next identified PAVs between the ‘Dangshansuli’ and ‘Bartlett’ genomes using ScanSV [25], with ‘Bartlett’ as the reference genome against which ‘Dangshansuli’ was aligned. A total of 92,694 PAVs were identified (Additional file 2) and used as a set of known PAVs with which to judge the accuracy of insertions and deletions detected by the three software packages. More SVs that overlapped with PAVs were detected using ONT than PB-CLR sequencing data. Sniffles_v2 identified more overlapping SVs between the ‘Dangshansuli’ and ‘Bartlett’ genomes from ONT sequencing data than from PB-CLR. These demonstrated that Sniffles_v2 can detect more SV with higher accuracy.

**Fig. 4:**
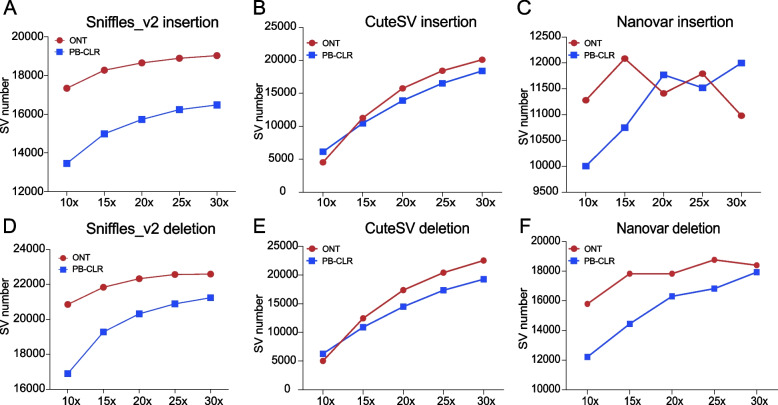
Insertions and deletions detected by three software packages (Sniffles_v2, CuteSV, and Nanovar) that overlapped with presence/absence variations (PAVs) between the pear cultivars ‘Bartlett’ and ‘Dangshansuli’. The red and blue lines represent SVs detected from ONT and PB-CLR sequencing data, respectively

As the sequencing depth increased, more overlapping SVs were detected by Sniffles_v2 and CuteSV, but not Nanovar (Fig. 4). However, the rate at which the number of overlapping SVs increased slowed as the sequencing depth increased (Additional file 3). Like CuteSV, Sniffles_v2 identified more SVs that overlapped with PAVs from ONT rather than PB-CLR sequencing data. Sniffles_v2 and CuteSV, detected a similar number of overlapping SVs at 30x sequencing depth using ONT data. Sniffles_v2 detected 19,024 insertions and 22,594 deletions while CuteSV reported 20,106 insertions and 22,543 deletions, both significantly greater than the 10,979 insertions and 18,403 deletions detected by Nanovar. Furthermore, Sniffles_v2 called more overlapping SVs than the other two software packages at low sequencing depth (10×) (Fig. 4). This suggested that the SVs detected by Sniffles_v2 had a higher confidence level.

Although Nanovar detected the highest number of SVs overall, it detected the fewest overlapping SVs. This implied that the SVs detected by Nanovar may have had a higher false positive rate. When the sequencing depth was 15×, Nanovar called the most overlapping insertions between two genomes, and the number of overlapping deletions did not significantly increase at higher sequencing coverage levels in the ONT data (Fig. 4C&F). Thus, 15× coverage may be sufficient for Nanovar, but the SVs detected with this program likely have a higher false positive rate than those called with Sniffles_v2 or CuteSV.

### Overlapping SVs among three software packages using PB-CLR and ONT sequencing data

To explore the similarities and differences of SVs detected with PB-CLR and ONT data, we counted the number of overlapping SVs from PB-CLR and ONT reads. SVs detected from ONT data accounted for a greater proportion of overlapping SVs than those detected from PB-CLR data (Additional file 4). When the sequencing depth was > 20×, CuteSV detected a higher number of overlapping insertions. Sniffles_v2 detected highest number of overlapping deletions.

The highest number of overlapping deletions was detected by Sniffles_v2 using ONT sequencing data at a sequencing depth of 30×. There was little difference between the three SV callers in detecting deletions. Slightly more overlapping deletions were called by the three software packages using ONT sequencing data compared to PB-CLR.

Nanovar identified the largest number of overlapping inversions and duplications out of the three software packages, whereas Sniffles_v2 and CuteSV identified the fewer. This suggested that although Sniffles_v2 and CuteSV performed well in detecting insertions, they did not detect inversions or duplications as well. Nanovar is therefore recommended for use when inversions or duplications are of particular importance.

**Fig. 5:**
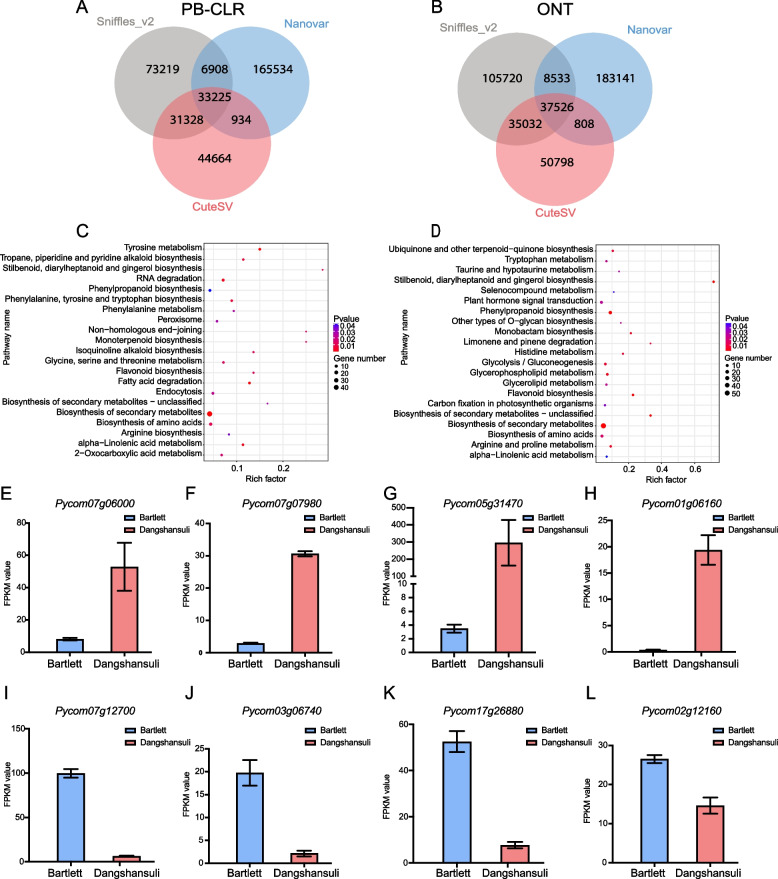
Expression levels of genes in regions that overlapped between SVs and presence/absence variations (PAVs) were compared between ‘Bartlett’ and ‘Dangshansuli’ pear samples to identify genes potentially associated with important agronomic traits. **A**, **B** Venn diagram showing SVs identified using three different SV detection programs on PB-CLR and ONT sequencing data, respectively. **C**, **D** KEGG pathway enrichment analysis of differentially expressed genes (DEGs) with SVs located in the promoter region. DEGs are shown as down-regulated (**C**) or up-regulated (**D**) in ‘Dangshansuli’ compared to ‘Bartlett’. **E**–**H** FPKM values of four candidate genes related to sugar, lignin, and fatty acid biosynthesis pathways

We next investigated the overlap between SVs detected by the three SV callers from PB-CLR and ONT sequencing data at 30× sequencing depth. SURVIVOR was used to merge VCF files from the three SV detectors. Performance was observed for four combinations: Sniffles_2 vs. CuteSV, Sniffles_2 vs. Nanovar, CuteSV vs. Nanovar, and Sniffles_2 vs. CuteSV vs. Nanovar (Additional file 5). The number of overlapping SVs identified with ONT was higher than those identified from PacBio sequencing data. From ONT sequencing data, there were a total of 37,526 SVs covering over 28 Mb in intra-chromosomes that overlapped among the three software packages, whereas there were 33,225 SVs covering over 18 Mb in intra-chromosomes that overlapped from the PB-CLR sequencing data (Fig. 5A&B). The overlapping SVs among three software packages using ONT sequencing were then combined with transcriptome profiles to mine genes associated with important agronomic traits that differ between ‘Bartlett’ and ‘Dangshansuli’ pears.

### Differentially expressed genes within SVs related to phenotypic differences between ‘Dangshansuli’ and ‘Bartlett’

We next investigated the relationship of overlapping SVs with annotated protein coding genes to assess their functional impact. There were 4427 SVs (12%) occurring in gene regions. However, 10,137 SVs (~ 27.6%) were located in the promoter region, whereas only 1798 SVs (4.9%) occurred in the coding sequence (CDS) region.

The genes within SVs were then annotated using InterProscan (IPR) and GO files; they were found to be associated with sugar metabolic pathways, fatty acid metabolism, alcohol dehydrogenase, lignin biosynthesis, and disease resistance (Fig. 5C&D, Additional file 6). Thirty-one sugar-related genes were identified, namely 20 genes involved in sugar metabolism and 11 sugar transporter genes (Additional file 6). A total of seven genes (*Pycom09g17070, Pycom10g02840, Pycom03g12440, Pycom01g17140, Pycom03g06740, Pycom11g06020* and *Pycom11g06030*) were annotated in pathways involved in metabolism of fatty acids, which are volatile compounds in pears. Seven alcohol dehydrogenase genes (*Pycom03g03290, Pycom07g06090, Pycom12g04030 Pycom08g09680, Pycom13g19000, Pycom13g22620 and Pycom13g22620*) were related to aromatic compound biosynthesis in pears. Interestingly, seven genes (*Pycom02g11000, Pycom03g01180, Pycom08g04540, Pycom04g09720, Pycom10g02150*, *Pycom10g18940 and Pycom15g31010*) were related to stone cell formation and two cyclin-like genes (*Pycom14g06300* and *Pycom12g06480*) were related to fruit size. These genes within SVs were submitted to the PlantTFDB website (http://planttfdb.gao-lab.org) to identify putative transcription factors; 141 plant transcription factors (TFs) were predicted, including 15 *MIKC_MADS* genes, 11 *MYB*s, nine *NAC*s, eight *bHLH*s, seven *bZIP*s, seven *HD-ZIP*s, seven *M-type_MADS*, seven *TALE*s, and six *WRKY*s (Additional file 7).

Differentially expressed genes (DEGs) were then identified in the flesh of ‘Bartlett’ and ‘Dangshansuli’ fruits collected 60 days after cross-pollination (DACP). ‘Bartlett’ was used as the reference genome. A total of 6465 genes were up-regulated and 6402 were down-regulated in ‘Dangshansuli’. Of those, 631 up-regulated genes and 702 down-regulated genes were present within SVs. These genes were then annotated using GO and KEGG, and transcription factors were predicted. Fifty-three of the DEGs within SVs were predicted to be transcription factors, with 32 up-regulated and 21 down-regulated in ‘Dangshansuli’. Furthermore, genes with SVs in their promoter regions were also predicted; 32 of these genes were up-regulated and 55 were down-regulated in ‘Dangshansuli’ (Additional file 7).

SVs occurring in promoter regions were further investigated. We annotated SVs located in promoters and compared that list with the DEGs between ‘Bartlett’ and ‘Dangshansuli’. In ‘Bartlett’, fifteen sugar-related genes were identified (Additional file 6). *Pycom06g04890* was expressed at an extremely high level in ‘Bartlett’. *Pycom14g06300,* which was associated with fruit size, highly expressed in ‘Bartlett’. *Pycom12g05120*, *Pycom03g06740, Pycom07g12700*, and *Pycom07g20170* were also highly expressed, and were all related to fatty acid metabolism (Additional file 6). In ‘Dangshansuli’ pear, DEGs with SVs in the promoter regions were mainly related to three important traits: lignin, sugar metabolism, and fatty acid metabolism. Fourteen peroxidase genes, 18 sugar-related genes, and 12 genes associated with lignin were also highly expressed in ‘Dangshansuli’. These candidate genes were then submitted to the PlantTFDB website to predict TFs. Several important TFs for fruit growth were present, such as *MYBs*, *MIKC_MADS*s, and *bHLH*s (Additional file 7). The FPKM of 8 genes selected from Additional files 6 and 7 were shown in Fig. 5E-L.

## Discussion

The goal of this study was to detect SVs in the pear genome with high accuracy using long-read sequencing data. To facilitate future study of SVs, we established a workflow for SV detection based on the tools evaluated in this study (Additional file 8). First, we compared the performance of Minimap2, NGMLR, LRA and Winnowmap2. Second, we compared the ability of three software packages to detect SVs from PB-CLR and ONT sequencing data at different sequencing depths. Third, we identified PAVs between the ‘Bartlett’ and ‘Dangshansuli’ genomes and counted the SVs that overlapped with PAVs. Fourth, SURVIVOR was used to merge SVs detected by all three SV-detecting programs and the shared SVs were annotated. Finally, we mined the genes within SVs using genomic and transcriptomic data to identify genes that may contribute to differences in agronomic traits between the ‘Bartlett’ and ‘Dangshansuli’ pear cultivars.

Significant challenges remain in detecting SVs in plant genomes, especially in polyploid species. Many programs that have previously been developed cannot accurately locate SVs, particularly multiple types of SVs, at low sequencing depths. Although SV detector software has iteratively improved over time, genome analyses, particularly SV studies, benefit from continued advances in sequencing technologies, computational algorithms, and reduced sequencing costs [4]. Higher sequencing depth is more conducive to detecting SVs with greater accuracy and specificity. Moreover, compared with read length and sequencing error, sequencing depth might be the most influential factor affecting the performance of SV calling. Using long reads (average 20 kb in length) with low error rates (7.5–10%), almost all SV callers perform well when the sequencing depth reaches 20× [26]. While for short reads, sequencing depth of 50× is appropriate for SV detection in the pear genome [22].

Single-molecule sequencing strategies generate contiguous reads that are tens to hundreds of kilobases long, which can greatly improve read alignment and allow more direct detection of SVs [27]. It was reported that SV detection in the human genome was approximately three times more sensitive with PacBio long reads than with short reads [28]. In the human genome, the use of ONT sequencing data resulted in the discovery of similar numbers of SVs as PacBio sequencing data, but ONT further allowed identification of small deletions (< 200 bp) that could not be detected with PacBio data [29, 30]. In the present study, PB-CLR and ONT long-read sequencing data were compared for the first time with respect to SV detection in the pear genome. In general, SV detection was superior from ONT compared to PB-CLR sequencing data. Based on the read mapping ratio, the number of SVs detected, the number of SVs identified by all three SV detector packages, and the number of SVs that overlapped with PAVs between ‘Bartlett’ and ‘Dangshansuli’, ONT sequencing data is recommended for future study of SVs.

The choice of SV detection software, sequencing costs, the types of SVs detected, and the accuracy of SV detection must be considered. In a previous study, Sniffles_v1 was reported to have increased accuracy compared to SVIM [22]. Newer algorithms have been developed since that time. Of the three SV detection software packages tested here, Sniffles_v2 had the highest sensitivity in detecting insertions and the highest accuracy in detecting all SVs, especially from ONT data. Additionally, we compared the Sniffles_v2 (v2.0.6) and Sniffles_v1 (v1.0.12) in SV detection with bam files generated by Minimap2. Sniffles_v2 showed better performance than Sniffles_v1 (Additional file 9). Nanovar had advantages in detecting SVs at low sequencing depth, but the SVs were detected with lower accuracy. There was little difference in the ability of SV callers to detect deletions. Where sequencing cost or the detection of duplications and inversions are concerns, Nanovar would be the recommended choice. Some similarity analysis was conducted in both studies. Duan et al. paid more attention to the advantages and disadvantages of each genotyping method using human genome and sequencing data [31]. We have some similar conclusion: CuteSV did better in detecting insertions and deletions.

Sequencing costs can limit the study of SVs, especially at a population-wide level [32]. The current sequencing price of ONT is cheaper than PB-CLR. To be more specific, the estimated cost per Gb for CLR generated from the latest platform of Sequel II is about $22, for ONT PromethION, it is about $19. For Sniffles_v2 and CuteSV, higher sequencing depths increased the number of SVs detected. In contrast, Nanovar was developed with low sequencing depth (8×) ONT data [24]. In the pear genome, Nanovar detected a large number of each type of SVs. Considering only the SVs that overlapped with PAVs between the two pear cultivar genomes, the number of insertions called by Nanovar from ONT sequencing data was highest at 15× coverage, and the number of deletions did not increase significantly from 10× to 15×. Therefore, Nanovar could be used to optimally detect SVs in the pear genome at 15× sequencing depth.

The length of SVs identified using ONT and PB-CLR data showed high difference. SURVIVOR was used to merge three vcf files from SV detection tools and the bed file was generated with location and types of SVs. The minimum size was set to 50 bp, and maximum size was set to 10,000 bp. ONT data detected an additional 10 Mb of overlapping SVs (Fig. 5), of which ~ 8 Mb more deletions were identified with ONT sequencing data compared to PB-CLR data (Additional file 10). The number of SVs detected using ONT data were more than using PB-CLR data (Fig. 2). Additionally, the length of deletions detected using PB-CLR and ONT data was significantly different (Fig. 3), which may have led to the differences (including 8 Mb deletions and other SVs) between ONT and PB-CLR data.

SVs represent an important component of genetic diversity in plants and have great impacts on phenotypic variation. Mining the genes contained within SVs can be conducive to identification of genetic differences that affect agronomic traits [4]. The ancient *Pyrus* lineage likely originated in the mountainous regions of Southwestern China [33]. It then spread east and west along the mountains, and geographic differences have led to the observed variations in biological characteristics of Asian and European pears [9]. The agronomic trait differences in Asian and European pear fruits include levels of sugars, acid, stone cells, and volatile compounds; flesh softness; and disease and stress resistance [9]. An SV occurred in the promoter of *Pycom05g31470 (PRX)* (Fig. 5G, Additional file 6)*,* which is reportedly involved in lignified secondary cell walls throughout stem development in *Arabidopsis thaliana* [34]. *Pycom05g31470* was here found to be highly expressed in the ‘Dangshansuli’ cultivar and low expression in ‘Bartlett’. It was reported that Asian pear has higher stone cell content and *Pycom05g31470* may associated with stone cell content [9].

It has been well established that many genes in a variety of plant tissues are regulated by key TFs. These TFs are typically classified into different families based on the conserved motifs that encode the DNA-binding domains [35]. SVs occurring in the promoter region may affect transcriptional binding sites, leading to differential expression of transcription factors. The *bHLH*, *EFR*, and *MYB* TF families are reportedly related to lignin, sugars, and fatty acids, respectively. *BSE1* (*Pycom02g12160*) was expressed at higher levels in ‘Bartlett’ than in ‘Dangshansuli’(Fig. 5L); in tomato (*Solanum lycopersicum*), *SlBES1* promotes fruit softening during fruit ripening and postharvest storage [36]. The regulatory mechanisms of these candidate TFs require further verification in multiple pear cultivars.

## Conclusion

Here, we conducted a comparison of SVs detected from PB-CLR and ONT sequencing data for two pear cultivars. Four mapping tools were compared, Minimap2, NGMLR, LRA and Winnowmap2. Minimap2 had a higher mapping ratio and was used in further analyses. Three SV callers (Sniffles_v2, CuteSV, and Nanovar) were tested on PB-CLR and ONT sequencing data at a range of sequencing depths. CuteSV had higher sensitivity in detecting insertions and deletions, whereas Nanovar performed better in detecting inversions and duplications using ONT sequencing data. The SVs detected by Sniffles_v2 had the highest accuracy using ONT sequencing data at 10× sequencing depth. However, the performance of Nanovar using ONT sequencing data showed that 15× coverage was sufficient to identify SVs in the pear genome. The SVs that were called by all three software packages were integrated with transcriptome data to identify genes related to agronomic traits that differ between the ‘Bartlett’ and ‘Dangshansuli’ pear cultivars. The candidate genes identified that are associated with levels of sugars, acid, stone cells, and aromatic compounds should be studied in more depth. The information uncovered here regarding SV detection from long reads, suitable sequencing depth, and integration of multi-omics data will promote and simplify the process of mining genes in SVs that affect agronomically important traits that differ between cultivars. This study provides significant insights into the selection of long-read sequencing platforms and SV detection programs that can be applied in future studies of crop genomes.

## Methods

### Pear materials

Young leaves were collected from ‘Dangshansuli’ pear trees in the germplasm orchard at Wenyangtian, Shandong Agricultural University. Young fruits were collected from ‘Bartlett’ and ‘Dangshansuli’ pear trees at Wenyangtian Modern Agricultural Industrial Park, Shandong Agricultural University 60 days after cross-pollination (DACP). Samples were screened at random for size uniformity and the absence of visible mechanical damage. Fruit and seed morphology were examined in a portion of samples, and the remainder were cut into pieces, immediately frozen in liquid nitrogen, and stored at − 80 °C prior to further analyses.

### Library construction and long-read sequencing

For ONT sequencing, DNA repair, end repair, and adapter ligation were conducted during library preparation. First, 49 μg of DNA from each sample was fragmented using the g-TUBE system (Covaris, Woburn, MA, USA). DNA repair was performed using the NEBNext FFPE DNA Repair Mix (New England Biolabs [NEB], Ipswich, MA, USA; M6630). End repair was conducted with the NEBNext End Repair/dA-Tailing Module (NEB, E7546). Adapter ligation was performed with the NEBNext Quick Ligation Module (NEB, E6056) and the Ligation Sequencing Kit 1D (ONT, SQK-LSK109). Only library fragments > 3 kb were retained for further analyses. DNA was purified between each step using Agencourt AMPure XP beads (Beckman Coulter, Brea, CA, USA). The flow cell chemistry was R9.4.1 and PromethION flow cell were used for sequencing DNA. The pear samples were multiplexed.

SMRTbell libraries were constructed following the standard PacBio protocol (Pacific Biosciences, Menlo Park, CA, USA) with 15 kb preparation solutions. The main steps for library preparation were as follows: (1) genomic DNA was sheared to ~ 20 kb; (2) removal of single-strand overhangs; (3) DNA damage repair; (4) end repair for blunt-end ligation; (5) blunt-end ligation; (6) template purification; (7) size selection with the BluePippin System; (8) sequencing primer annealing to the SMRTbell template; (9) sequencing polymerase binding to the SMRTbell template; and (10) sequencing preparation. For each sample, 3 μg of genomic DNA was sheared with a g-TUBE. The PacBioCLR library preparations were conducted with the SMRTbell Express Template Prep Kit 1.0 as instructed by the manufacturer. The CLR SMRTbell template–polymerase complexes were sequenced on a PacBioSequel instrument using the Sequel Sequencing Kit 3.0 with 6 Sequel™ SMRT® Cells 1 M v3, taking a 10-h movie per cell. Finally, the libraries were sequenced on a PacBio Sequel II platform using 2.0 chemistry.

### Read extraction to generate a range of sequencing depths

Using the program Seqtk (https://github.com/lh3/seqtk), the command ‘seqtk sample’ was used to randomly extract subsamples of cleaned reads at different depths. Long reads were sampled to depths of 10, 15, 20, 25, and 30× coverage.

### Read mapping

For PacBio reads, SMRTlink v6.0 (https://www.pacb.com/wp-content/uploads/) was used to filter out low quality reads. For ONT reads, Guppy (https://pypi.org/project/ont-pyguppy-client-lib/) was used for real-time base calling and Nanoplot (https://github.com/wdecoster/NanoPlot) was to count the raw reads. ‘Bartlett’ (v2) and ‘Dangshansuli’ (v1.1) genome assembly files were obtained from the Genome Database for Rosaceae (GDR) [37]. Minimap2 [17], NGMLR [18], LRA [19], Winnowmap2 [20] were used for read mapping. NGMLR was used to map long reads to the ‘Bartlett’ reference genome (parameters: ‘ngmlr -t 50 -r Bartlett.fa -q dangshansuli.fq -o dangshansu.sam’). The parameters used for Minimap2 were as follows: ‘minimap2 -ax map-pb bartlett.fasta -t 50 dangshan_pac.fq >dangshan_pac.sam’ and ‘minimap2 -ax -ax map-ont bartlett.fasta -t 50 dangshan_ONT.fq >dangshan_ONT.sam’. The long reads were mapped to ‘Bartlett’ genome using LRA need two steps: ‘lra index –CLR/ONT Bartlett.fa’ and ‘lra align –CLR/ONT –t 50 Bartlett.fa dangshansuli.fq –p s > dangshansuli.sam’. For Winnowmap2 aligner, *k* = 17 was founded in ‘Bartlett’ genome and ‘meryl count *k*=17 output merylDB Bartlett.fa’ was used to count. And then, ‘meyl print merylDB > repetitive_k17.txt’ and ‘winnowmap -k 17 -W repetitive_k17.txt -ax map-pb-clr -t 50 --MD bartlett.fa dangshansuli.fq > dangshansuli.sam’ were used to generate sam files. Aligned files in SAM format were converted to BAM format, then sorted using SAMtools (v1.9) [38].

Noteworthy, for ONT reads, the bam files generated from LRA or Winnowmap2 need ‘samtools calmd’ to generate ‘NM’ and ‘MD’ tag with sorted bam files.

### SV detection using three different programs

After mapping reads to the reference genome, SVs were identified from the processed BAM files. We compared the performance of Sniffles_v2 (v2.0.6) (https://github.com/fritzsedlazeck/Sniffles), CuteSV [23], and Nanovar [24] in detecting SVs between ‘Bartlett’ and ‘Dangshansuli’. All three programs were able to detect SVs from both PB-CLR and ONT sequencing data. Insertion positions were determined by extracting the sequences 100 bp upstream and downstream of the predicted location. Insertion lengths were determined from the “SVLEN” values in the output SV-VCF files.

The default parameters were used for Sniffles_v2 were as follows: ‘sniffles --input bam_file --vcf SV.vcf’, respectively. The parameters used for CuteSV were: ‘cuteSV dangshan.sorted.bam bartlett.fasta cutesv.vcf --max_cluster_bias_INS 100 --diff_ratio_merging_INS 0.3 --max_cluster_bias_DEL 200 --diff_ratio_merging_DEL 0.5 -t 30’. For Nanovar data, the following parameters were used for the PB-CLR sequencing data: ‘nanovar -x pacbio-clr dangshan_Pac.sorted.bam bartlett.fasta. /nanovar_work’; for the ONT sequencing data, the following parameters were used: ‘nanovar -x ont dangshan_ONT.sorted.bam bartlett.fasta. /nanovar_work’. For all three programs, the output files were in VCF format and included the chromosome number, position, SV type, and quality for each SV.

### PAV calling between ‘Bartlett’ and ‘Dangshansuli’ pear

‘Bartlett’ and ‘Dangshansuli’ genome files were obtained from GDR [37]. ScanPAV [25] was used to detect PAVs between ‘Bartlett’ and ‘Dangshansuli’ pear genomes using the default parameters. The ‘Dangshansuli’ genome was aligned using ‘Bartlett’ as the reference genome. The PAVs identified are shown in Additional file 2.

### SV merging and analysis

SURVIVOR (v1.0.7) [39], a tool kit for assessing SVs with multiple modules, was used in this analysis. The minimum SV length was set to 50 bp. SURVIVOR was used to filter and combine the calls from VCF files with Sniffles_v2, CuteSV and Nanovar. It works to convert the method-specific output formats to a VCF format and SVs were filtered out if they were unique to one of the three VCF files. In the end, SURVIVOR produced one VCF file containing the so filtered calls and provided an extended bed file to report the locations of the simulated SVs.

### Transcriptome profile analysis

Transcriptome profiles were generated from ‘Bartlett’ and ‘Dangshansuli’ fruits at 60 DACP with three replicates. The raw RNA-seq data was processed using trim_galore (https://github.com/FelixKrueger/TrimGalore) (‘-q 25 --phred33 --length 36 -e 0.1 --stringency 3 –paired’) to obtain clean data by removing low-quality reads. The Q20, Q30, and GC content of the clean data were also calculated. The following analyses were based on the cleaned data. The index of the reference genome was built using hisat2-build and clean reads were aligned to ‘Bartlett’ (*P. communis*) CDS regions using hisat2 (‘hisat2 -x bartlett -p 10 -1 fq1 -2 fq2 –S sample.sam’) [40]. Gene expression was calculated using the expected number of Fragments Per Kilobase of transcript per Millions of base pairs sequenced (FPKM) method, which simultaneously considers the effect of sequencing depth and gene length to normalize read counts [41]. Differential gene expression analysis was performed with the DESeq2 R package [42]. Genes were considered to be significantly differentially expressed between pear varieties at *p* < 0.05.

### Gene ontology and KEGG pathway enrichment

To explore the distribution of functional categories and biochemical pathways of the SV targets, GO term [43] (www.geneontology.org) and KEGG pathway [44] enrichment analyses were performed using Blast2GO [45] and KOBAS 2.0 [46] (http://kobas.cbi.pku.edu.cn/). All GO categories and KEGG pathways were screened at a threshold of *p* < 0.05.

## Supplementary Information

**Additional file 1.** SVs detected by Sniffles_v2, CuteSV, and Nanovar after mapping with Minimap2, NGMLR, LRA and Winnowmap2.

**Additional file 2.** List of presence/absence variations (PAVs) between the pear cultivars ‘Bartlett’ and ‘Dangshansuli’.

**Additional file 3.** SVs detected by Sniffles_v2, CuteSV, and Nanovar that overlapped with PAVs at a range of sequencing depths.

**Additional file 4.** SVs detected using both PB-CLR and ONT sequencing data at a range of sequencing depths.

**Additional file 5.** The number of SVs detected by Sniffles_v2, CuteSV, and Nanovar and merged by SURVIVOR.

**Additional file 6.** Annotation of genes within SVs detected by all three SV callers.

**Additional file 7.** Differentially expressed TFs within SVs.

**Additional file 8.** The overall workflow for SV detection using three SV-calling programs on PB-CLR and ONT sequencing data.

**Additional file 9.** The number of SVs detected by Sniffles_v1 and Sniffles_v2 (SV length > 50 bp).

**Additional file 10.** The length of different types of overlapping SVs from three SV detection tools.

## Data Availability

All raw sequence data generated in this study has been deposited in NCBI under BioProject accession number: PRJNA852344 and SRA accession number: SRR19843133, SRR19843134, SRR19843141, SRR19843142, SRR19843143, SRR19843144, SRR19843145 and SRR19843146. All other supporting data are included as additional files.

## Abbreviations

BAM file: Binary version of SAM file
BCF: Binary Counterpart Data
FPKM: Fragments Per Kilobase of transcript per Millions of base pairs
GO: Gene Ontology
InDel: Insertion or deletion
IPR: InterProScan
KEGG: Kyoto Encyclopedia of Genes and Genomes
ONT: Oxford Nanopore Technologies
Pacbio-CLR: Continuous long reads from Pacific Biosciences
PB-CLR: Continuous long reads from Pacific Biosciences
SAM: Sequence alignment/map format
SV: Structural variant
TF: Transcription factors
VCF: Variant Call Format
