# Supplementary material for: Comparison of structural variants detected by PacBio-CLR and ONT sequencing in pear
Source: BMC Genomics. 2022 Dec 14;23:830. doi: 10.1186/s12864-022-09074-7 (PMC9753399; doi:10.1186/s12864-022-09074-7)
Supplement: Supplementary file 8 — Additional file 8. The overall workflow for SV detection using three SV-calling programs on PB-CLR and ONT sequencing data. [file 12864_2022_9074_MOESM8_ESM.pdf]

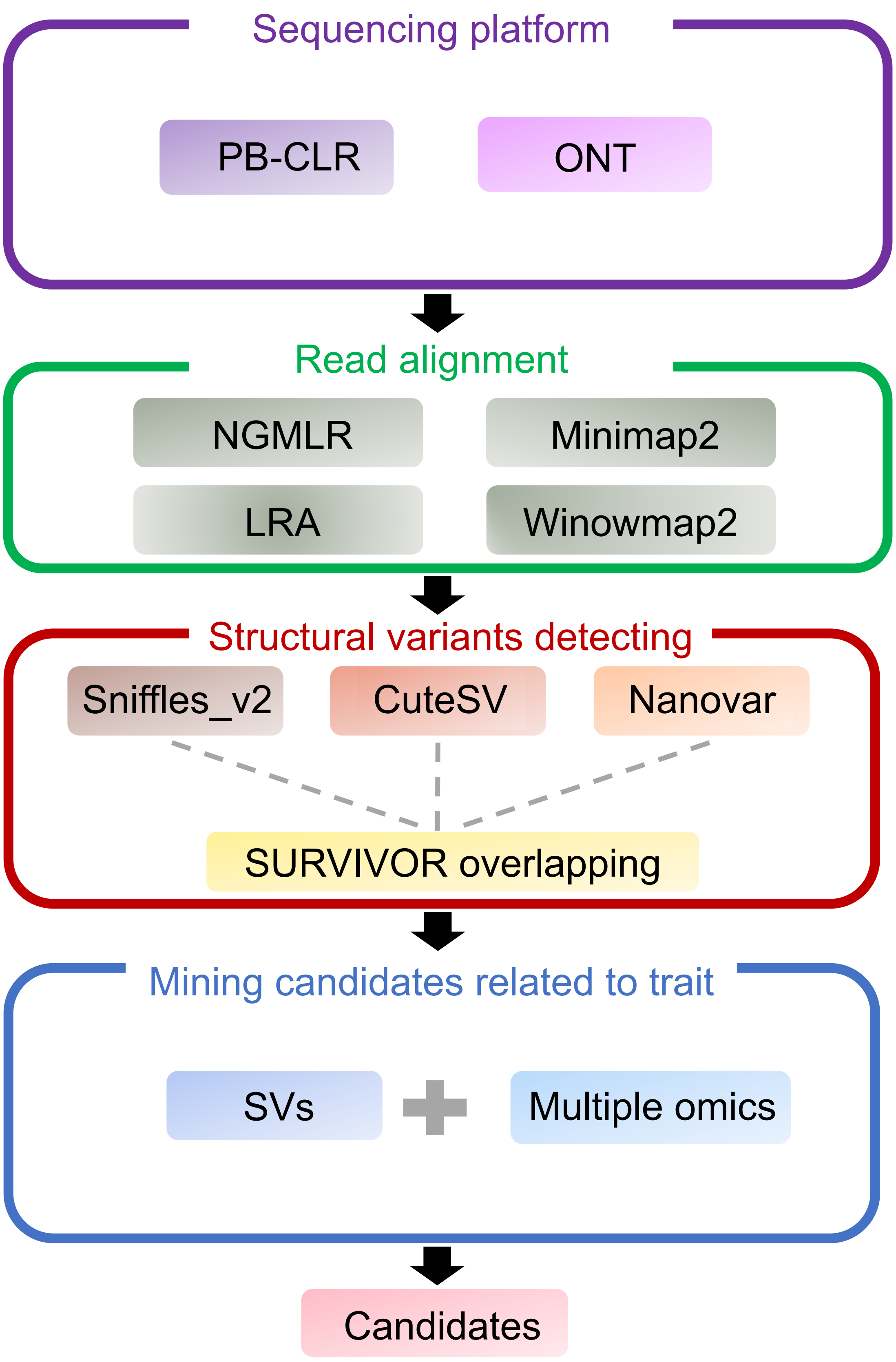

Additional file 8. The overall workflow for SV detection using three SV-calling programs on PB-CLR and ONT sequencing data.
